# Factors associated with the effectiveness of opioids for dyspnea in hospitalized patients with heart failure: a retrospective, multicenter, observational study

**DOI:** 10.1186/s40780-025-00523-5

**Published:** 2025-12-09

**Authors:** Shoichi Yoshikai, Yasushi Moriya, Tomoyuki Yamada, Junichi Higuchi, Emi Goto, Masami Nishihara, Akira Ashida, Kenji Ikeda

**Affiliations:** 1 Department of Clinical Pharmacy Research and Education, Graduate School of Pharmaceutical Sciences, The University of Osaka, 1-6 Yamadaoka, Suita, Osaka 565-0871 Japan; 2 Department of Pharmacy, Ohara Healthcare Foundation Kurashiki Central Hospital, 1-1-1 Miwa, Kurashiki, Okayama 710-8602 Japan; 3 Department of Pharmacy, Osaka Medical and Pharmaceutical University Hospital, 2-7 Daigaku-machi, Takatsuki, Osaka 569-8686 Japan

**Keywords:** Dyspnea, Heart failure, Opioid, Palliative care

## Abstract

**Background:**

The decision to administer opioids for dyspnea caused by heart failure should be made on an individual basis. However, factors associated with the effectiveness of opioids remain unclear, and information that could guide this decision is lacking. This study aimed to explore factors associated with the effectiveness of opioids to relieve dyspnea caused by heart failure.

**Methods:**

This retrospective, multicenter, observational study included patients 18 years of age or older at the time of admission who received opioid administration (morphine, oxycodone, or hydromorphone) for dyspnea relief but did not receive invasive ventilatory support during hospitalization. Data were collected from the medical records.

**Results:**

A total of 129 cases (30 ineffective cases, 80 effective cases, and 19 cases with missing dyspnea assessment data) were collected. The multivariable logistic regression analysis results indicated that the odds ratios of the New York Heart Association classification, number of diuretics used, and oxygen flow rate associated with the presence or absence of dyspnea improvement attributable to opioid administration were 0.496 (95% confidence interval, 0.254–0.967), 1.506 (95% confidence interval, 1.131–2.007), and 0.974 (95% confidence interval, 0.923–1.027), respectively. Trends in odds ratios for both the number of diuretics used and New York Heart Association classification were preserved in the multivariable logistic regression model that included factors that potentially influence dyspnea (*p* < 0.05).

**Conclusion:**

The effectiveness of opioids for relieving dyspnea caused by heart failure in hospitalized patients was positively associated with the number of diuretics used and negatively associated with the New York Heart Association classification.

**Supplementary Information:**

The online version contains supplementary material available at 10.1186/s40780-025-00523-5.

## Background

The number of patients with heart failure worldwide is rapidly increasing as the population ages [1, 2]. Because the trajectory of heart failure is associated with a decline in the quality of life, the need for palliative care for heart failure is recognized [3]. Management of dyspnea, which is the most common symptom of heart failure [4], is a priority of palliative care. Although only a few treatments that relieve dyspnea in patients with heart failure are available, the 2021 European Society of Cardiology guidelines state that opioids may be effective [5]. Among opioids, morphine [6, 7] and oxycodone [8] can effectively relieve dyspnea, and hydromorphone [9] has the potential to effectively relieve dyspnea caused by heart failure. However, some studies have concluded that opioids are ineffective for such cases [10]; therefore, the conclusions regarding the ability of opioids to provide effective dyspnea relief are inconsistent. Furthermore, opioid use for dyspnea with heart failure may be associated with increased adverse events [11]. Therefore, opioids should not be administered routinely [5]; instead, they should be considered on an individual basis [12]. However, information that could guide individualized decision-making is lacking. The decision to administer opioids is considered a component of specialized palliative care for patients with heart failure; furthermore, this decision is more frequently made for patients who are hospitalized in institutions with a well-established palliative care infrastructure [6, 8, 9].

Patients who are likely to experience minimal toxicity and the maximum effectiveness of opioids must be identified before opioid administration can be individualized on a case-by-case basis [13]; however, factors that identify such patients have not yet been clarified. Therefore, we conducted an exploratory study to determine which factors are associated with the ability of opioids to effectively relieve dyspnea caused by heart failure to promote their appropriate use for such patients.

## Methods

### Survey participants

Surveys were conducted from January 1, 2010 to September 30, 2021 at The University of Osaka Hospital, from January 1, 2017 to May 1, 2022 at Kurashiki Central Hospital, and from January 1, 2014 to October 31, 2022 at Osaka Medical and Pharmaceutical University Hospital. These survey periods were dependent on when we were able to extract medical records at each institution. The University of Osaka Hospital and Osaka Medical and Pharmaceutical University Hospital are Special Functioning Hospitals, whereas Kurashiki Central Hospital is a Regional Medical Care Support Hospital [14]. Special Functioning Hospital and Regional Medical Care Support Hospital classifications indicate that these institutions are equipped with more advanced medical resources compared to those available at general hospitals. Furthermore, The University of Osaka Hospital, Osaka Medical and Pharmaceutical University Hospital, and Kurashiki Central Hospital have more than 900 beds and cardiology and palliative care departments. These institutions were selected as study sites because of their potential to enroll sufficient subjects. The inclusion criteria were admission to the aforementioned hospitals and medical records that confirmed opioid administration to relieve dyspnea caused by heart failure during hospitalization. The purpose of opioid use was verified using records prepared by the medical practitioners who managed the patient. The presence or absence of opioid prescriptions was verified using the prescription history. Each data collector had more than 10 years of clinical experience. When a decision could not be made independently, data were recorded following a discussion among the data collectors. Opioids were defined as morphine hydrochloride, oxycodone hydrochloride hydrate, or hydromorphone hydrochloride, as listed in the National Health Insurance drug price list. The exclusion criteria were age younger than 18 years at the time of hospitalization and invasive artificial ventilation use during the opioid administration period.

### Survey items

Patient background information and physiological data at the time of admission included sex, age (years), New York Heart Association (NYHA) classification, height (cm), weight (kg), body mass index (kg/m²), left ventricular ejection fraction (LVEF; %), and average oxygen flow rate during opioid administration (L/day). Additionally, we investigated the following classifications based on the LVEF: heart failure with preserved ejection fraction (LVEF ≥ 50%); heart failure with mid-range ejection fraction (LVEF ≥ 40% to < 50%); and heart failure with reduced ejection fraction (LVEF < 40%). The oxygen concentration (fraction of inspiratory oxygen [FiO_2_]; %) was converted to the oxygen flow rate using a conversion table [15].

Laboratory data obtained at the time of admission included the levels of albumin (Alb; g/dL), brain natriuretic peptide (pg/mL), serum creatinine (mg/dL), aspartate aminotransferase (IU/L), alanine aminotransferase (IU/L), sodium (mEq/L), and potassium (mEq/L), as well as the estimated glomerular filtration rate (mL/min).

The opioid type, drug administration form, drug administration route, dosage (mg/day), duration of administration (from initiation to discontinuation; days), and types of concomitant medications during opioid administration (Additional file 1) were investigated. The dose was converted to an intravenous morphine-equivalent dose according to the National Comprehensive Cancer Network guidelines [16].

The average respiratory rate (breaths/min), heart rate (beats/min), and saturation of percutaneous oxygen (SpO_2_; %) were measured before opioid administration, on the first day of administration, and on the third day of administration. Data regarding the respiratory status, respiratory distress, and adverse events were also collected.

### Outcomes

The primary outcome was dyspnea improvement after opioid administration. The preadministration period ranged from the hospitalization day to the day before the start of opioid administration. The postadministration period ranged from the initial opioid administration day to the opioid discontinuation day. The period from the initiation of opioid exposure to discontinuation, i.e., the postadministration period, corresponds to both the duration administered and the follow-up duration. Dyspnea improvement was defined as an improvement of ≥ 1 point on the numerical rating scale, ≥ 10 mm on the visual analog scale, ≥ 1 point on the Borg dyspnea scale, or ≥ 1 point on the Japanese version of the Support Team Assessment Schedule (STAS-J).

The numerical rating scale, visual analog scale, and Borg dyspnea scale scores were adopted from the evaluation results documented in the medical records created by medical practitioners who assessed the patients. In accordance with a previous study, the STAS-J score was assessed by the data collector based on the information in the medical records prepared by medical practitioners [17]. Before performing the assessment, all data collectors thoroughly reviewed the STAS-J Scoring Manual [18] to ensure the consistency and accuracy of their assessments. Symptom assessments for patients receiving palliative care are fundamentally subjective. Therefore, in this study, assessments performed using subjective evaluation tools such as the numerical rating scale, visual analog scale, and Borg dyspnea scale were prioritized; STAS-J assessments were performed only when other subjective evaluation tool data were not available in the medical records. Definitions used for the numerical rating scale, visual analog scale, and Borg dyspnea scale were based on the minimal clinically important difference [13]. The Support Team Assessment Schedule (STAS) was designed to objectively assess the symptom severity of patients receiving palliative care. The reliability and validity of the STAS-J are equivalent to those of the original STAS [19]. The STAS-J has an ordinal scale structure with scores ranging from 0 to 4 according to item-specific definitions. The definition of improvement according to the STAS-J was based on a difference of ≥1 point, which was considered the minimal clinically important difference. When multiple dyspnea assessments were conducted within the pretreatment and posttreatment periods, the value closest to that at the time of treatment initiation was adopted. When multiple dyspnea assessments were performed on the same day, the median value was used.

Cases with dyspnea improvement were defined as the effectiveness group. Cases without dyspnea improvement were defined as the ineffectiveness group. Cases for which improvement in dyspnea could not be determined because of insufficient information in the medical records were defined as the missing group.

### Statistical analysis and analytical processing

Survey items were described using summary statistics and presented as frequencies and percentages or means ± standard deviation. For continuous survey items regarding patient background information, physiological data, and laboratory data, the absolute standardized differences (ASDs) between the effectiveness group and the combined ineffectiveness group and missing group were calculated. For survey items with high missing cases, the distributions of other survey items were assessed separately for cases with missing data and for cases without missing data.

The missing group and ineffectiveness group were considered as one group during the statistical analysis. The missing group did not meet the predefined criteria for opioid effectiveness and was therefore classified as the ineffectiveness group in the analysis. Missing values were imputed using the multiple imputation method. Then, a multivariable logistic regression analysis was conducted to obtain odds ratios and 95% confidence intervals for each explanatory variable. The significance level was set at *p* < 0.05. The dependent variable in the multivariable logistic regression model was the presence or absence of an improvement in dyspnea. Based on previous studies, explanatory variables were preferentially selected as factors potentially associated with the status of or improvement in dyspnea. The NYHA classification was used to categorize heart failure severity and assign categories 1 to 4 to cases based on subjective symptoms and the degree of limitation in daily physical activity. NYHA category 4 represents more severe symptoms, such as dyspnea, that increase the likelihood of opioid administration. Previously, opioids have been administered to patients with NYHA category 3 or category 4 heart failure [6, 7]. Oxygen therapy is commonly administered for dyspnea associated with heart failure [20, 21]; therefore, the NYHA classification and oxygen flow rate were included as explanatory variables. The number of explanatory variables included in the multivariable logistic regression model was determined according to the rule of including one explanatory variable for every 10 cases in the smaller of the two outcome groups (i.e., the group with fewer cumulative cases of the effectiveness group or the combined ineffectiveness and missing group) [22]. Including an excessive number of explanatory variables in the model beyond this criterion can result in overfitting. Explanatory variable selection based on univariate significance testing is dependent on the sample size and often results in the inclusion of explanatory variables with mixed causal relationships, leading to interpretation difficulties [23]. Therefore, ASDs, which are not dependent on the sample size, were used for explanatory variable selection, and survey items with an ASD >0.5 were selected. When considering concomitant medications as candidate explanatory variables, only those with a usage rate of ≥20% in the combined ineffective group and missing group were selected because of concerns that medications with extremely low usage rates may lack statistical stability or clinical relevance and could contribute to model overfitting [24]. As sensitivity analyses, analyses excluding the missing group and those incorporating institutions as nominal variables during multiple imputation were performed. Statistical analyses were performed using R software (R version 4.4.1; The R Foundation, Vienna, Austria; https://www.r-project.org/). The multiple imputation method was performed using the mice package.

## Results

**Fig. 1:**
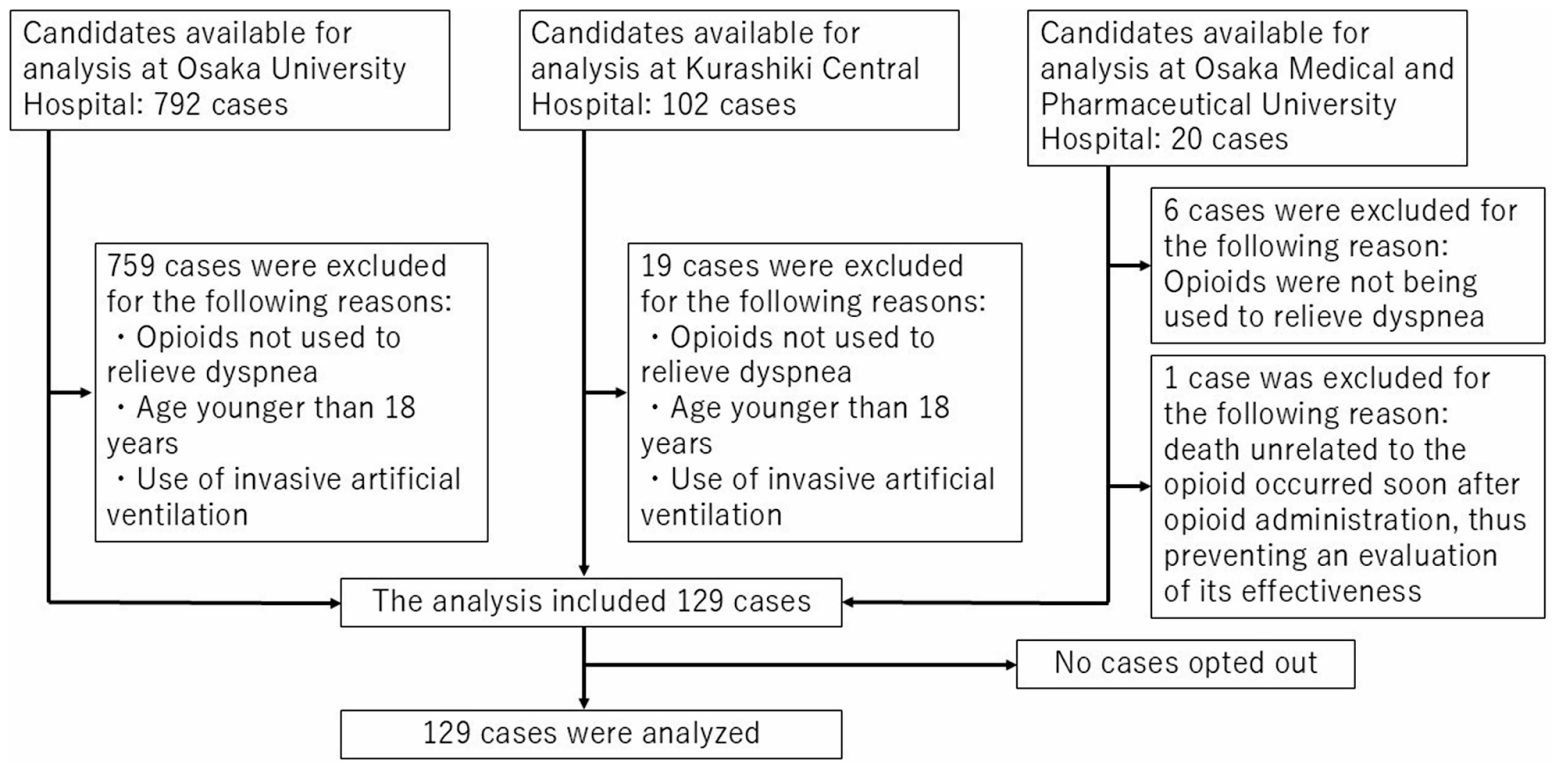
Study flowchart showing the case selection process

A total of 129 cases, including 30 (23%) in the ineffectiveness group, 80 (62%) in the effectiveness group, and 19 (15%) in the missing group, were collected (Fig. 1). At The University of Osaka Hospital, opioids used during surgery are recorded as prescription data. Consequently, during data extraction based on the presence of an opioid prescription during hospitalization, a larger cohort than that at the other institutions was identified. In 18 cases of the missing group, the reason was that the　patient’s self-reported severity of dyspnea could not be obtained due to clinical deterioration. In one case of the missing group, the patient was discharged immediately after the initiation of medication, making evaluation difficult. Regarding patient background information, physiological data, and laboratory data, the proportion of cases classified as NYHA category 4 was highest in the ineffectiveness group, whereas that classified as NYHA category 3 was highest in the effectiveness group. The ASD for Alb exceeded 0.5 (Table 1). Due to the high number of cases with missing NYHA classification, the distributions of cases with and without missing NYHA classification were assessed for the survey items in Table 1 and institution information. Kurashiki Central Hospital was found to have a higher number of cases with missing NYHA classification (Additional file 2).

**Table 1:** Summary of patient background Information, physiological data, and laboratory data

Survey Items	Ineffectiveness Group (*N* = 30)	Effectiveness Group (*N* = 80)	Missing Group (*N* = 19)	ASD Between the Effectiveness Group and Combined Ineffectiveness Group and Missing Group	
Sex				-	
Female	11 (37%)	41 (51%)	10 (53%)		
Male	19 (63%)	39 (49%)	9 (47%)		
Age (years)	81 ± 9	79 ± 13	82 ± 9	0.2	
NYHA classification				-	
2	2 (12%)	3 (5.7%)	0 (0%)		
3	2 (12%)	27 (51%)	1 (25%)		
4	13 (76%)	23 (43%)	3 (75%)		
Unknown	13	27	15		
Height (cm)	156 ± 11	157 ± 11	154 ± 10	0.18	
Unknown	2	5	0		
Weight (kg)	54 ± 12	53 ± 14	50 ± 14	0.03	
Unknown	2	3	0		
BMI (kg/m^2^)	22.1 ± 4.0	20.6 ± 5.8	20.9 ± 3.7	0.21	
Unknown	2	5	0		
Laboratory data					
Alb (g/dL)	2.72 ± 0.59	2.95 ± 0.85	2.39 ± 0.46	0.61	
Unknown	0	1	0		
BNP (pg/mL)	1349 ± 1544	1812 ± 2015	1501 ± 1596	0.25	
Unknown	5	10	4		
LVEF (%)	35 ± 17	34 ± 16	38 ± 16	0.13	
HFpEF	7 (27%)	18 (24%)	7 (37%)		
HFmrEF	4 (15%)	13 (17%)	2 (11%)		
HFrEF	15 (58%)	45 (59%)	10 (53%)		
Unknown	4	4	0		
Scr (mg/dL)	2.37 ± 1.60	2.30 ± 1.49	2.17 ± 1.53	0.08	
Unknown	0	1	0		
eGFR (mL/min)	31 ± 24	30 ± 22	34 ± 27	0.22	
Unknown	0	1	0		
AST (IU/L)	125 ± 311	39 ± 38	45 ± 64	0.34	
Unknown	0	1	0		
ALT (IU/L)	91 ± 253	31 ± 47	33 ± 60	0.32	
Unknown	0	1	0		
Na (mEq/L)	140 ± 11	137 ± 8	139 ± 7	0.21	
Unknown	0	1	0		
K (mEq/L)	4.27 ± 0.57	4.41 ± 0.82	4.44 ± 0.66	0.09	
Unknown	0	1	0		
Oxygen flow (L/day)	7.4 ± 11.0	4.8 ± 5.6	7.2 ± 8.5	0.32	
Unknown	2	0	0		

Data are presented as n (%) or mean ± standard deviation. n (%) represents the proportion of cases in each group

Alb, albumin; ALT, alanine aminotransferase; ASD, absolute standardized difference; AST, aspartate aminotransferase; BMI, body mass index; BNP, brain natriuretic peptide; eGFR, estimated glomerular filtration rate; HFmrEF, heart failure with mid-range ejection fraction; HFpEF, heart failure with preserved ejection fraction; HFrEF, heart failure with reduced ejection fraction; K, potassium; Na, sodium; NYHA, New York Heart Association; Scr, serum creatinine

Most dyspnea assessments were based on the STAS-J, and the mean assessment date was 3 days after opioid initiation (Table 2). Regarding concomitant medications, the ASD for diuretics and antiarrhythmic drugs exceeded 0.5. The total number of patients who used diuretics in the ineffective group and missing group exceeded 10 (Table 3).

**Table 2:** Summary of dyspnea assessment results

Dyspnea Assessment	Ineffectiveness Group (*N* = 30)	Effectiveness Group (*N* = 80)	Missing Group (*N* = 19)
Assessment method			
NRS/VAS/Borg dyspnea scales	1 (3.3%)	1 (1.3%)	-
STAS-J	29 (97%)	79 (99%)	-
Unknown	-	-	19 (100%)
Assessment day	3 ± 2	3 ± 2	-

Data are presented as n (%) or mean ± standard deviation. n (%) represents the proportion of cases in each group

NRS, numerical rating scale; STAS-J, Japanese version of the Support Team Assessment Schedule; VAS, visual analog scale

**Table 3:** Use of concomitant medications

Medications Used (*n*)	Ineffectiveness Group (*N* = 30)	Effectiveness Group (*N* = 80)	Missing Group (*N* = 19)		ASD Between the Effectiveness Group and Combined Ineffectiveness Group and Missing Group
Diuretics					0.57
0	9 (30%)	11 (14%)	9 (47%)		
1	13 (43%)	20 (25%)	3 (16%)		
2	3 (10%)	23 (29%)	3 (16%)		
3	2 (6.7%)	13 (16%)	1 (5.3%)		
4	2 (6.7%)	6 (7.5%)	3 (16%)		
5	1 (3.3%)	5 (6.3%)	0 (0%)		
6	0 (0%)	1 (1.3%)	0 (0%)		
7	0 (0%)	1 (1.3%)	0 (0%)		
Vasodilators					0.31
0	28 (93%)	66 (83%)	18 (95%)		
1	1 (3.3%)	11 (14%)	1 (5.3%)		
2	1 (3.3%)	3 (3.8%)	0 (0%)		
Cardiotonic agents/pressor agents					0.21
0	10 (33%)	21 (26%)	7 (37%)		
1	9 (30%)	24 (30%)	6 (32%)		
2	9 (30%)	22 (28%)	2 (11%)		
3	1 (3.3%)	11 (14%)	4 (21%)		
4	1 (3.3%)	2 (2.5%)	0 (0%)		
β-blockers					0.09
0	29 (96.7%)	66 (82%)	13 (68%)		
1	1 (3.3%)	14 (18%)	6 (32%)		
Alpha β-blockers					0.23
0	27 (90%)	70 (87%)	19 (100%)		
1	3 (10%)	10 (13%)	0 (0%)		
ACE inhibitors/ARBs					0.28
0	28 (93.3%)	71 (89%)	19 (100%)		
1	2 (6.7%)	9 (11%)	0 (0%)		
Antiarrhythmics					0.54
0	29 (96.7%)	63 (79%)	18 (94.7%)		
1	1 (3.3%)	17 (21%)	1 (5.3%)		
Sodium-glucose cotransporter 2 inhibitors					0.05
0	29 (96.7%)	76 (95%)	18 (94.7%)		
1	1 (3.3%)	4 (5.0%)	1 (5.3%)		
If channel inhibitors (channel blockers)					-
0	30 (100%)	80 (100%)	19 (100%)		
Angiotensin receptor neprilysin inhibitors					-
0	30 (100%)	80 (100%)	19 (100%)		
Soluble guanylate cyclase stimulators					-
0	30 (100%)	80 (100%)	19 (100%)		
Others					-
0	30 (100%)	80 (100%)	19 (100%)		
Hypnotics/sedatives					0.12
0	19 (63%)	44 (55%)	12 (63%)		
1	6 (20%)	23 (29%)	6 (32%)		
2	3 (10%)	10 (13%)	1 (5.3%)		
3	1 (3.3%)	3 (3.8%)	0 (0%)		
4	1 (3.3%)	0 (0%)	0 (0%)		
Antipsychotics					0.01
0	22 (73%)	64 (80%)	16 (84%)		
1	8 (27%)	15 (19%)	3 (16%)		
3	0 (0%)	1 (1.3%)	0 (0%)		
Antidepressants					
0	28 (93%)	74 (93%)	18 (95%)		
1	2 (6.7%)	5 (6.3%)	1 (5.3%)		
3	0 (0%)	1 (1.3%)	0 (0%)		

Data are presented as n (%). n (%) represents the proportion of cases in each group

ACE, angiotensin-converting enzyme; ARB, angiotensin II receptor blocker; ASD, absolute standardized difference

Amiodarone was the most frequently administered antiarrhythmic drug (Additional file 3). Morphine was the most commonly administered opioid. Drugs were most commonly administered as an injection. The intravenous route was the most common route of drug administration. Opioid doses (intravenous morphine-equivalent dose) were similar among the three groups. The duration of opioid administration (days administered) was shortest in the ineffectiveness group (Table 4).

**Table 4:** Use of opioids

Survey Items	Ineffectiveness Group (*N* = 30)	Effectiveness Group (*N* = 80)	Missing Group (*N* = 19)
First opioid administered			
Morphine	25 (83%)	70 (88%)	19 (100%)
Oxycodone	0 (0%)	2 (2.5%)	0 (0%)
Hydromorphone	5 (17%)	8 (10%)	0 (0%)
Administration form			
Injection	27 (90%)	76 (95%)	18 (95%)
Orally administered internal agents	3 (10%)	4 (5.0%)	1 (5.3%)
Administration route			
Oral administration	3 (10%)	4 (5.0%)	1 (5.3%)
Intravenous injection	27 (90%)	73 (91%)	18 (95%)
Others	0 (0%)	3 (3.8%)	0 (0%)
Dose (mg/day)	8 ± 7	7 ± 6	6 ± 3
Duration administered (days)	7 ± 8	13 ± 14	8 ± 10
Reason for discontinuation			
Dyspnea improvement	0 (0%)	16 (20%)	0 (0%)
Lack of dyspnea improvement	0 (0%)	1 (1.3%)	0 (0%)
Death	26 (87%)	53 (66%)	18 (95%)
Discharge from hospital	0 (0%)	4 (5.0%)	1 (5.3%)
Adverse events	2 (6.7%)	4 (5.0%)	0 (0%)
Others	2 (6.7%)	2 (2.5%)	0 (0%)

Data are presented as n (%) or mean ± standard deviation. n (%) represents the proportion of cases in each group. Duration administered corresponds to both the postadministration period and the follow-up duration

The respiratory rates, heart rates, and SpO_2_ of the ineffectiveness and effectiveness groups from the time before opioid administration until administration day 3 were similar (Additional file 4). Additionally, respiratory depression did not occur after opioid administration. Regarding adverse events, high frequencies of nausea, constipation, and somnolence were observed (Additional file 5).

### Selection of explanatory variables incorporated in the multivariable logistic regression analysis

Eighty cases in the effectiveness group and 49 cases in the combined ineffectiveness and missing group were collected. A maximum of four explanatory variables could be included in the multivariable logistic regression model. Among the concomitant medications, the number of diuretics used had an ASD > 0.5, and the proportion of diuretics use in the combined ineffective group and missing data group was ≥ 20% (i.e., ≥ 10 cases). Therefore, the NYHA classification, oxygen flow rate, and number of diuretics used were selected as explanatory variables for the multivariable logistic regression analysis (model 1). No known studies have demonstrated an association between the number of antiarrhythmic drugs and dyspnea or between Alb levels and dyspnea in patients with heart failure. However, an ASD > 0.5 was observed. The Alb level and number of antiarrhythmic drugs used may have influenced the results of model 1. Therefore, a model (model 2) including the number of antiarrhythmic drugs used as a fourth variable in model 1 and a model (model 3) including Alb as a fourth variable in model 1 were also analyzed.

### Multivariable logistic regression analysis results

The results of the multivariable logistic regression analysis in which the presence or absence of dyspnea improvement caused by opioids was the dependent variable indicated that the odds ratio of the number of diuretics was 1.506 (*p* = 0.005). This finding suggested that the use of several diuretics is associated with increased opioid effectiveness. The odds ratio of the NYHA classification was 0.496 (*p* = 0.040), indicating that patients with lower NYHA classifications were more likely to benefit from opioid use. In model 2, which included the number of antiarrhythmic drugs used as an explanatory variable, and in model 3, which included Alb as an explanatory variable, the trends of the odds ratios of the NYHA classification and number of diuretics used were maintained (*p* < 0.05). In model 2, the odds ratio of the number of antiarrhythmic drugs used was 4.827 (*p* = 0.049), indicating that opioids were more effective when antiarrhythmic drugs were also used. In model 3, the odds ratio of Alb was 2.092 (*p* = 0.038), suggesting that the effectiveness of opioids was greater when Alb levels were higher (Table 5). As sensitivity analyses, analyses excluding the missing group and those incorporating institutions as nominal variables in multiple imputation were performed, yielding similar results (Additional file 6 and 7).

**Table 5:** Multivariable logistic regression analysis results

		Model 1				Model 2				Model 3					
Explanatory Variable		OR	95%CI	*P*		OR	95%CI	*P*		OR	95%CI			*P*	
NYHA classification		0.496	0.254–0.967	0.040		0.485	0.246–0.960	0.038		0.501	0.253–0.993			0.048	
Diuretics (n)		1.506	1.131–2.007	0.005		1.442	1.076–1.933	0.014		1.455	1.086–1.948			0.012	
Oxygen flow		0.974	0.923–1.027	0.326		0.981	0.930–1.034	0.466		0.983	0.932–1.037			0.532	
Antiarrhythmics (n)						4.827	1.005–23.178	0.049							
Albumin										2.092	1.041–4.205			0.038	

CI, confidence interval; NYHA, New York Heart Association; OR, odds ratio

## Discussion

### Factors associated with the ability of opioids to effectively relieve dyspnea in patients with heart failure

All multivariable logistic regression analysis models revealed a significant association between the number of diuretics used and the ability of opioids to effectively relieve dyspnea in patients with heart failure. In other words, even after adjusting for factors that may influence dyspnea in patients with heart failure and covariates with an ASD > 0.5, the number of diuretic medications used appeared to be associated with the effectiveness of opioids.

Congestive symptoms that do not improve with monotherapy diuretic regimens may improve with the addition of other diuretics [25, 26]. Combination therapy comprising diuretics is recommended to resolve severe congestive symptoms [27]. Therefore, patients with heart failure who received intensive combination therapy comprising diuretics likely had persistent pulmonary congestion similar to that reported in previous studies [25, 26]. Opioids inhibit the secretion of vasopressin, which is an antidiuretic hormone, through opioid µ and κ receptors, resulting in a diuretic effect [28]. Heart failure severity is associated with increased vasopressin secretion [29]. Therefore, one possible explanation for the results of this study is that opioids exert a diuretic effect by inhibiting the secretion of vasopressin, which is elevated in patients with heart failure and severe congestive symptoms. This diuretic effect may have acted synergistically with the intensively administered diuretics, thereby contributing to dyspnea improvement. This explanation is supported by studies that reported that morphine increases diuresis in patients with heart failure [30] and the combination of morphine and diuretics enhances the diuretic effect compared to that of diuretics alone in patients with high-altitude pulmonary edema [31].

The results indicated that opioid effectiveness was more likely to be achieved when antiarrhythmic drugs were also administered. Among antiarrhythmic drugs, amiodarone was most commonly used. According to the package insert, amiodarone is contraindicated for patients with severe respiratory failure in Japan [32]. Therefore, these results may reflect situations in which many patients in the effectiveness group were judged by their attending physicians to have an adequate respiratory status for amiodarone administration. However, the 95% confidence interval for antiarrhythmic drugs was wider than that for the other explanatory variables; this may have been attributable to their low usage rate. Because of this uncertainty, caution is warranted when interpreting these results.

Significant differences in the NYHA classification were observed in all models. The results indicated that higher NYHA classification categories were associated with lower opioid effectiveness. Previous studies have reported that a better Eastern Cooperative Oncology Group Performance Status (ECOG PS) is associated with increased opioid effectiveness [33]. A moderate negative correlation between the category of NYHA classification and score of Karnofsky Performance Status (KPS) has been observed, and a negative correlation exists between the score of ECOG PS and score of KPS [34, 35]. Therefore, the NYHA classification was considered to be correlated with the ECOG PS, consistent with previous findings. Furthermore, the results showed that higher Alb levels were associated with increased opioid effectiveness. Alb is positively correlated with the ECOG PS and associated with in-hospital mortality of patients with heart failure [36, 37]. Thus, a higher Alb level reflects a better overall clinical condition, and patients with a preserved general status may be more likely to experience opioid effectiveness, in agreement with previous studies.

To consider the clinical applicability of these findings, it is necessary to present a detailed profile of patients with heart failure in good general condition and to discuss the potential for dyspnea to serve as an indication for opioid use in such patients. Patients with heart failure typically follow a clinical trajectory in which they first experience predisposing conditions such as hypertension before developing acute heart failure. Thereafter, they undergo repeated exacerbations and remissions of heart failure, with a gradual decline in cardiac function, ultimately progressing to refractory or end-stage disease [3]. The NYHA classification is an objective measure derived from patients’ subjective symptoms and limitations in physical activity; however, it does not necessarily provide an accurate reflection of subjective indicators, such as the perceived intensity of dyspnea [38]. In other words, even patients classified as NYHA category 2 or 3 may experience dyspnea of sufficient severity to warrant opioid treatment. Therefore, patients with heart failure who are in good general condition have not yet reached the terminal stage, and it can be interpreted that opioids may be effective for managing the severe dyspnea experienced by such patients.

One possible pathophysiological explanation for the findings observed in this study is as follows. One symptom in patients who have heart failure and are presumed to have poor systemic condition, such as those in NYHA category 4, is hepatomegaly associated with systemic congestion. When hepatomegaly is present, elevated levels of liver enzymes, including AST and ALT, as well as decreased Alb levels, may be observed [39]. Many opioids are metabolized in the liver; therefore, hepatic enlargement may reduce their hepatic metabolism. morphine undergoes hepatic glucuronidation to form morphine-6-glucuronide, a metabolite exhibiting greater pharmacological activity than morphine [40]. Consequently, a decrease in the production of this potent metabolite may reduce the effect for dyspnea. In the ineffectiveness group, elevated AST and ALT levels may indicate the presence of hepatomegaly. Moreover, the majority of opioids administered in this study were morphine. In other words, the production of the highly active metabolite morphine-6-glucuronide may have been reduced in patients in the ineffectiveness group. Therefore, the association between NYHA classification or Alb levels and opioid effectiveness may result from reduced hepatic metabolism of morphine among patients with heart failure whose systemic condition has deteriorated, such as those exhibiting higher NYHA classifications or lower Alb levels, in whom hepatic enlargement associated with systemic congestion occurs.

Missing NYHA classifications were frequently observed at Kurashiki Central Hospital (Additional file 2). The missing data were thought to be attributable to differences in evaluation record formats across institutions. However, based on the results of the sensitivity analysis (Additional file 7), the impact of missing NYHA classifications on the analysis results is considered minimal.

Routine opioid use for dyspnea caused by heart failure is not recommended [5], and a prolonged study period was necessary to accumulate sufficient cases. Throughout the study period, guideline descriptions regarding pharmacotherapy for acute heart failure, diuretics were the mainstay of treatment for hospitalized patients with acute heart failure, vasodilators were administered to patients with preserved blood pressure, and chronic heart failure medications (such as β-blockers) were recommended only after the patient’s condition had stabilized [5, 41–46]. During opioid administration, diuretics and vasodilators were commonly used and β-blockers were infrequently used. This medication use pattern was consistent with the guideline recommendations and suggested a generally uniform treatment approach. Furthermore, guideline descriptions regarding opioids, primarily morphine, shifted from their use in the initial management of acute heart failure to their use in palliative care [5, 41–46]. However, these guidelines have consistently recommended considering opioids for patients experiencing severe dyspnea. Given the unpredictable prognosis of patients with heart failure [47], it is presumed that clinicians consider opioid use whenever they judge that a patient is experiencing severe dyspnea, regardless of the patient’s overall condition or circumstances. In Japan, the national health insurance system ensures universal coverage during this study period, and socioeconomic factors have minimal influence on treatment [48]. Accordingly, the impact of the prolonged study period, as well as the influence of the timing of assessment in individual cases, was considered minimal. A systematic review of the efficacy of opioids for dyspnea caused by heart failure [11] investigated the literature regarding patients with stable heart failure symptoms who received outpatient treatment and concluded that opioid administration was not effective and posed the risk of increased adverse events. These findings suggested that opioids should be used only as a last resort after all other options have failed or in emergency situations. However, these findings were not derived from hospitalized patients who received opioids for emergency indications or severe dyspnea. In contrast, the present study focused on these patients. Furthermore, the systematic review [11] did not provide information regarding predictors of opioid efficacy. Therefore, the present study sought to address this gap in the existing literature.

### Strengths and limitations

The present study collected more cases than those included in previous studies of opioid use to relieve dyspnea caused by heart failure. Furthermore, although many studies of patients with stabilized heart failure symptoms and those who underwent outpatient treatment have been reported, very few studies of hospitalized patients with heart failure are available [6]. Additionally, the present study provided substantial information regarding opioid administration for hospitalized patients with heart failure. However, this study had some limitations. The exploratory investigation was based on retrospective observational research. Although characteristics associated with the effectiveness of opioids for the specified target population were identified, all the information necessary to confirm these associations with certainty was not obtained. For example, direct indicators of congestion, such as the composite congestion score [49], were not available. Additionally, because there is no standardized method of converting the doses of all diuretics to a single indicator, dosage information could not be clarified. The reasons for the concomitant use of diuretics were not documented in the medical records; therefore, they could not be clearly determined. However, because diuretics may contribute to reduced congestion, their potential impact on the results was considered minimal. Outcomes were predominantly based on STAS-J scores. Subjective evaluations are rarely performed, probably because of their difficulty [6] attributable to the cognitive function or condition of patients and lack of familiarity with palliative care among medical practitioners in the cardiology field [50]. To the best of the authors’ knowledge, no studies have investigated the correlation between STAS or STAS-J and subjective measures such as the numerical rating scale. However, studies evaluating the effect of opioids on dyspnea in patients with lung cancer employed both the numerical rating scale and STAS to assess efficacy, and both measures demonstrated an effect following opioid administration [51]. That is, the directionality of subjective assessment tools, such as the numerical rating scale, generally aligns with that of the STAS-J, suggesting a low potential for bias. However, the potential impact of employing different evaluation methods simultaneously cannot be dismissed. To overcome these limitations, data from prospective registry studies with the same objective as that of the present study should be analyzed.

## Conclusions

Both the number of diuretics used and NYHA classification were significantly associated with the effectiveness of opioids for dyspnea caused by heart failure. Therefore, patients who receive multiple diuretics are more likely to experience effective relief from dyspnea with opioid administration, whereas patients with higher NYHA classification categories are less likely to benefit from opioid treatment. This information can be used when making individualized decisions regarding opioid administration for patients with heart failure; additionally, it can be used to avoid adverse events caused by unnecessary opioid administration. Further research should be performed to confirm the results of this study.

## Supplementary Information

Below is the link to the electronic supplementary material.

Supplementary Material 1

Supplementary Material 2

Supplementary Material 3

Supplementary Material 4

Supplementary Material 5

Supplementary Material 6

Supplementary Material 7

## Data Availability

The datasets generated and analyzed during the current study are not publicly available due to privacy concerns and institutional policy but are available from the corresponding author on reasonable request.

## Abbreviations

Alb: Albumin
ASD: Absolute standardized difference
ECOG PS: Eastern Cooperative Oncology Group Performance Status
FiO_2_: Fraction of inspiratory oxygen
KPS: Karnofsky Performance Status
LVEF: Left ventricular ejection fraction
NYHA: New York Heart Association
SpO_2_: Saturation of percutaneous oxygen
STAS: Support Team Assessment Schedule
STAS-J: Japanese version of the Support Team Assessment Schedule
