# Supplementary material for: Factors associated with the effectiveness of opioids for dyspnea in hospitalized patients with heart failure: a retrospective, multicenter, observational study
Source: J Pharm Health Care Sci. 2025 Dec 9;12:6. doi: 10.1186/s40780-025-00523-5 (PMC12802230; doi:10.1186/s40780-025-00523-5)
Supplement: Supplementary file 1 — Supplementary Material 1 [file 40780_2025_523_MOESM1_ESM.docx]

Additional file 1. Concomitant Medications

| Classification | Corresponding Medications |
| --- | --- |
| Diuretics | Thiazide diuretics  Combination preparation of thiazide diuretics and ARBs  Combination preparation of thiazide diuretics, ARBs, and calcium channel blockers  Thiazide-like diuretics  Loop diuretics  Potassium-sparing diuretics and aldosterone antagonists  Vasopressin V2 receptor antagonists  Carbonic anhydrase inhibitors |
| Vasodilators | Vasodilators and antiplatelet agents (dipyridamole)  Antihypertensive agents (hydralazine)  Nitrates  Cardiovascular function-improving agents (trapidil)  Cardiac and renal disease treatment agents (dilazep)  Anti-ischemic agents (trimetazidine)  Nicorandil  α-Human atrial natriuretic polypeptide (carperitide) |
| Cardiotonic agents/pressor agents | Catecholamine inotropic agents  Digitalis  PDE inhibitors  Adenylyl cyclase activators (colforsin daropate)  Calcium sensitizers (pimobendan) |
| Beta-blockers | Beta-blockers |
| Alpha beta-blockers | Alpha beta-blockers |
| ACE inhibitors/ARBs | ACE inhibitors  ARBs  Combination preparation of ARBs and calcium channel blockers |
| Antiarrhythmics | Antiarrhythmics |
| Sodium-glucose cotransporter 2 inhibitors | Sodium-glucose cotransporter 2 inhibitors  Combination preparation of sodium-glucose cotransporter 2 inhibitors and DPP4 inhibitors |
| If channel inhibitors (channel blockers) | If channel inhibitors (channel blockers) |
| Angiotensin receptor neprilysin inhibitors | Angiotensin receptor neprilysin inhibitors |
| Soluble guanylate cyclase stimulators | Soluble guanylate cyclase stimulators |
| Others | ATP, ubiquinone, taurine |
| Hypnotics/sedatives | α2-Adrenergic agonist (dexmedetomidine)  Melatonin receptor agonists  Orexin receptor antagonists  Barbiturate hypnotic sedatives  General anesthesia/sedation agents (propofol)  Benzodiazepine hypnotic sedatives  Nonbenzodiazepine hypnotic sedatives |
| Antipsychotics | Antipsychotics |
| Antidepressants | Tricyclic antidepressants  Tetracyclic antidepressants  Noradrenergic and specific serotonergic antidepressants  Selective norepinephrine reuptake inhibitors  Selective serotonin reuptake inhibitors  Serotonin antagonist and reuptake inhibitors  Serotonin-norepinephrine reuptake inhibitors |

ACE, angiotensin-converting enzyme; ARB, angiotensin II receptor blocker; DPP4, dipeptidyl peptidase-4; PDE, phosphodiesterase
