# Supplementary material for: Factors associated with the effectiveness of opioids for dyspnea in hospitalized patients with heart failure: a retrospective, multicenter, observational study
Source: J Pharm Health Care Sci. 2025 Dec 9;12:6. doi: 10.1186/s40780-025-00523-5 (PMC12802230; doi:10.1186/s40780-025-00523-5)
Supplement: Supplementary file 2 — Supplementary Material 2 [file 40780_2025_523_MOESM2_ESM.docx]

Additional file 2. Distribution of survey items by the presence or absence of missing NYHA classification

| Survey items | NYHA Categories 2 - 4 N ＝ 74 | NYHA missing  N = 55 |
| --- | --- | --- |
| Institution |  |  |
| The University of Osaka Hospital | 27 (36%) | 6 (11%) |
| Kurashiki Central Hospital | 34 (46%) | 49 (89%) |
| Osaka Medical and Pharmaceutical University Hospital | 13 (18%) | 0 (0%) |
| Sex |  |  |
| Female | 32 (43%) | 30 (55%) |
| Male | 42 (57%) | 25 (45%) |
| Age (years) | 79 ± 11 | 82 ± 12 |
| Height (cm) | 157 ± 11 | 155 ± 10 |
| Unknown | 1 | 6 |
| Weight (kg) | 53 ± 13 | 52 ± 13 |
| Unknown | 1 | 4 |
| BMI (kg/m^2^) | 20.8 ± 5.3 | 21.4 ± 5.0 |
| Unknown | 1 | 6 |
|  |  |  |
| Laboratory data |  |  |
| Alb (g/dL) | 2.97 ± 0.85 | 2.61 ± 0.58 |
| Unknown | 1 | 0 |
| BNP (pg/mL) | 1,727 ± 1,815 | 1,570 ± 1,945 |
| Unknown | 8 | 11 |
| LVEF (%) | 32 ± 17 | 39 ± 15 |
| HFpEF | 15 (20%) | 17 (31%) |
| HFmrEF | 6 (8%) | 13 (24%) |
| HFrEF | 50 (68%) | 20 (36%) |
| Unknown | 3 | 5 |
| Scr (mg/dL) | 2.29 ± 1.47 | 2.31 ± 1.58 |
| Unknown | 1 | 0 |
| eGFR (mL/min) | 30 ± 22 | 31 ± 25 |
| Unknown | 1 | 0 |
| AST (IU/L) | 50 ± 75 | 74 ± 225 |
| Unknown | 1 | 0 |
| ALT (IU/L) | 39 ± 66 | 54 ± 185 |
| Unknown | 1 | 0 |
| Na (mEq/L) | 137 ± 9 | 139 ± 7 |
| Unknown | 1 | 0 |
| K (mEq/L) | 4.39 ± 0.72 | 4.36 ± 0.78 |
| Unknown | 1 | 0 |
| Oxygen flow (L/day) | 5.7 ± 8.8 | 5.7 ± 5.7 |
| Unknown | 1 | 1 |
| Data are presented as n (%) or mean ± standard deviation. n (%) represents the proportion of cases in each group.  Alb, albumin; ALT, alanine aminotransferase; ASD, absolute standardized difference; AST, aspartate aminotransferase; BMI, body mass index; BNP, brain natriuretic peptide; eGFR, estimated glomerular filtration rate; HFmrEF, heart failure with mid-range ejection fraction; HFpEF, heart failure with preserved ejection fraction; HFrEF, heart failure with reduced ejection fraction; K, potassium; Na, sodium; NYHA, New York Heart Association; Scr, serum creatinine. | | |
