# Supplementary material for: Factors associated with the effectiveness of opioids for dyspnea in hospitalized patients with heart failure: a retrospective, multicenter, observational study
Source: J Pharm Health Care Sci. 2025 Dec 9;12:6. doi: 10.1186/s40780-025-00523-5 (PMC12802230; doi:10.1186/s40780-025-00523-5)
Supplement: Supplementary file 3 — Supplementary Material 3 [file 40780_2025_523_MOESM3_ESM.docx]

Additional file 3. Detailed Breakdown of Antiarrhythmic Drug Use

| Antiarrhythmic Drug | Ineffectiveness Group (N = 1) | Effectiveness Group (N = 17) | Missing Group (N = 1) |
| --- | --- | --- | --- |
| Amiodarone | 1 (100%) | 11 (64.7%) | 1 (100%) |
| Mexiletine |  | 2 (11.8%) |  |
| Sotalol |  | 2 (11.8%) |  |
| Verapamil |  | 1 (5.9%) |  |
| Diltiazem |  | 1 (5.9%) |  |
| N represents the number of cases for which antiarrhythmic drugs were used. Data are presented as n (%). n (%) represents the proportion of cases in each group. | | | |
