# Supplementary material for: Factors associated with the effectiveness of opioids for dyspnea in hospitalized patients with heart failure: a retrospective, multicenter, observational study
Source: J Pharm Health Care Sci. 2025 Dec 9;12:6. doi: 10.1186/s40780-025-00523-5 (PMC12802230; doi:10.1186/s40780-025-00523-5)
Supplement: Supplementary file 4 — Supplementary Material 4 [file 40780_2025_523_MOESM4_ESM.docx]

Additional file 4. Respiratory Rate, Heart Rate, and SpO_2_ Levels from the Day Before Opioid Administration to Day 3 of Opioid Administration

| Characteristics | Ineffectiveness Group (N = 30) | Effectiveness Group (N = 80) | Missing Group (N = 19) |
| --- | --- | --- | --- |
| Respiratory rate before administration (breaths/min) | 24 ± 8 | 24 ± 8 | 20 ± 7 |
| Unknown | 18 | 50 | 8 |
| Heart rate before administration (beats/min) | 91 ± 14 | 89 ± 18 | 99 ± 23 |
| Unknown | 1 | 0 | 1 |
| SpO_2_ before administration (%) | 96.02 ± 3.30 | 96.29 ± 2.15 | 95.31 ± 2.91 |
| Unknown | 1 | 1 | 1 |
| Respiratory rate on day 1 (/min) | 24 ± 7 | 22 ± 6 | 25 ± 7 |
| Unknown | 10 | 30 | 4 |
| Heart rate on day 1 (/min) | 92 ± 15 | 91 ± 18 | 103 ± 27 |
| Unknown | 0 | 0 | 1 |
| SpO_2_ on day 1 (%) | 94.55 ± 5.50 | 96.23 ± 2.62 | 95.56 ± 2.41 |
| Unknown | 1 | 1 | 1 |
| Respiratory rate on day 3 (breaths/min) | 20.6 ± 4.2 | 18.9 ± 4.7 | 17.2 ± 4.0 |
| Unknown | 15 | 29 | 9 |
| Heart rate on day 3 (beats/min) | 89 ± 11 | 91 ± 18 | 107 ± 30 |
| Unknown | 10 | 6 | 6 |
| SpO_2_ on day 3 (%) | 95.87 ± 3.01 | 96.09 ± 2.25 | 96.19 ± 2.61 |
| Unknown | 10 | 6 | 6 |
| Data are presented as n or mean ± standard deviation. n (%) represents the proportion of cases in each group.  SpO_2_, saturation of percutaneous oxygen. | | | |
