# Supplementary material for: Factors associated with the effectiveness of opioids for dyspnea in hospitalized patients with heart failure: a retrospective, multicenter, observational study
Source: J Pharm Health Care Sci. 2025 Dec 9;12:6. doi: 10.1186/s40780-025-00523-5 (PMC12802230; doi:10.1186/s40780-025-00523-5)
Supplement: Supplementary file 5 — Supplementary Material 5 [file 40780_2025_523_MOESM5_ESM.docx]

Additional file 5. Incidence of Adverse Events

| Adverse Events | Ineffectiveness Group (N = 30) | Effectiveness Group (N = 80) | Missing Group (N = 19) |
| --- | --- | --- | --- |
| Nausea | 1 (3.3%) | 7 (8.8%) | 1 (5.3%) |
| Constipation | 0 (0%) | 6 (7.5%) | 0 (0%) |
| Delirium | 2 (6.7%) | 4 (5.0%) | 0 (0%) |
| Somnolence | 1 (3.3%) | 6 (7.5%) | 2 (11%) |
| Vomiting | 1 (3.3%) | 2 (2.5%) | 0 (0%) |
| Oversedation | 0 (0%) | 1 (1.3%) | 0 (0%) |
| Disorientation | 0 (0%) | 2 (2.5%) | 1 (5.3%) |
| Dizziness | 0 (0%) | 1 (1.3%) | 0 (0%) |
| Agitation | 1 (3.3%) | 0 (0%) | 0 (0%) |
| Psychological dependence | 0 (0%) | 1 (1.3%) | 0 (0%) |
| Clouded consciousness | 0 (0%) | 1 (1.3%) | 0 (0%) |
| Falls | 0 (0%) | 1 (1.3%) | 0 (0%) |
| Data are presented as n (%). n (%) represents the proportion of cases in each group. | | | |
