# Supplementary material for: Factors associated with the effectiveness of opioids for dyspnea in hospitalized patients with heart failure: a retrospective, multicenter, observational study
Source: J Pharm Health Care Sci. 2025 Dec 9;12:6. doi: 10.1186/s40780-025-00523-5 (PMC12802230; doi:10.1186/s40780-025-00523-5)
Supplement: Supplementary file 6 — Supplementary Material 6 [file 40780_2025_523_MOESM6_ESM.docx]

Additional file 6. Results of multivariate logistic regression analysis after excluding the missing group

|  | Model 1 | | | | Model 2 | | | | Model 3 | | | |
| --- | --- | --- | --- | --- | --- | --- | --- | --- | --- | --- | --- | --- |
| Explanatory Variable | | OR | 95%CI | *P* | | OR | 95%CI | *P* | | OR | 95%CI | *P* |
| NYHA classification | | 0.479 | 0.215–1.064 | 0.071 | | 0.484 | 0.216–1.088 | 0.079 | | 0.484 | 0.217–1.081 | 0.077 |
| Diuretics (n) | | 1.592 | 1.105–2.293 | 0.013 | | 1.500 | 1.034–2.175 | 0.033 | | 1.557 | 1.077–2.252 | 0.019 |
| Oxygen flow | | 0.978 | 0.925–1.034 | 0.432 | | 0.983 | 0.930–1.040 | 0.554 | | 0.981 | 0.928–1.038 | 0.508 |
| Antiarrhythmics (n) | |  |  |  | | 5.318 | 0.642–44.057 | 0.121 | |  |  |  |
| Albumin | |  |  |  | |  |  |  | | 1.354 | 0.666–2.755 | 0.402 |
| CI, confidence interval; NYHA, New York Heart Association; OR, odds ratio. | | | | | | | | | | | | |
