# Supplementary material for: Factors associated with the effectiveness of opioids for dyspnea in hospitalized patients with heart failure: a retrospective, multicenter, observational study
Source: J Pharm Health Care Sci. 2025 Dec 9;12:6. doi: 10.1186/s40780-025-00523-5 (PMC12802230; doi:10.1186/s40780-025-00523-5)
Supplement: Supplementary file 7 — Supplementary Material 7 [file 40780_2025_523_MOESM7_ESM.docx]

Additional file 7. Results of multivariate logistic regression analysis after incorporating the institution into the dataset and performing multiple imputation

|  | Model 1 | | | | Model 2 | | | | Model 3 | | | |
| --- | --- | --- | --- | --- | --- | --- | --- | --- | --- | --- | --- | --- |
| Explanatory Variable | | OR | 95%CI | *P* | | OR | 95%CI | *P* | | OR | 95%CI | *P* |
| NYHA classification | | 0.476 | 0.241–0.941 | 0.033 | | 0.471 | 0.235–0.944 | 0.034 | | 0.489 | 0.243–0.980 | 0.044 |
| Diuretics (n) | | 1.497 | 1.126–1.991 | 0.005 | | 1.435 | 1.073–1.920 | 0.015 | | 1.447 | 1.082–1.936 | 0.013 |
| Oxygen flow | | 0.974 | 0.923–1.027 | 0.334 | | 0.981 | 0.931–1.034 | 0.472 | | 0.983 | 0.933–1.037 | 0.536 |
| Antiarrhythmics (n) | |  |  |  | | 4.747 | 0.987–22.829 | 0.052 | |  |  |  |
| Albumin | |  |  |  | |  |  |  | | 2.074 | 1.031–4.173 | 0.041 |
| CI, confidence interval; NYHA, New York Heart Association; OR, odds ratio. | | | | | | | | | | | | |
